# The characteristics of the breaststroke pullout in elite swimming

**DOI:** 10.3389/fspor.2022.963578

**Published:** 2022-08-23

**Authors:** Carla McCabe, Emma Mosscrop, Ryan Hodierne, Elaine Tor

**Affiliations:** ^1^School of Sport, Sport and Exercise Sciences Research Institute, Ulster University, Belfast, United Kingdom; ^2^Faculty of Science and Engineering, Manchester Metropolitan University, Manchester, United Kingdom; ^3^British Swimming, Sports Science and Sports Medicine, Loughborough, United Kingdom; ^4^New South Wales Institute of Sport, Performance Support, Sydney, NSW, Australia; ^5^Forethought Pty Ltd., Melbourne, VIC, Australia

**Keywords:** start, turn, race-analysis, competition, fly-kick placement, underwater, breaststroke

## Abstract

Since the rule change permitting the inclusion of one dolphin kick during the underwater breaststroke pullout phase following a swim start or turn, there has been an emergence of several different pullout techniques adopted by elite swimmers. The aim of this study was to characterize the underwater breaststroke pullout technique trends and to assess the effectiveness of each technique as utilized by elite male and female swimmers. The sample included 60 swimmers (*n* = 26 male, *n* = 34 female) competing across the 50, 100, and 200 m long-course breaststroke final races from the World Championships 2015, 2017, 2019 and Olympic Games 2016. An above-water camera was used to identify and measure the different phases of the underwater pullout techniques, which was found to be a highly accurate methodological approach (ICC = 0.97). From the 150 trials analyzed, three different pullout techniques were identified: the Fly-Kick First technique, the Combined technique and the Pull-Down First technique. Although the most common underwater pullout technique utilized by elite competitive breaststroke swimmers was the Combined technique (*n* = 71), followed by the Fly-Kick First technique (*n* = 65) and the Pull-Down First technique (*n* = 14), it was observed that technical selection deviates according to gender. This indicates that male and female swimmers should not be coached adhering to the same technical model. This study found no significant difference in terms of performance outcome with respect to each of these techniques, indicating that technique selection should be guided by one's individual preference. It was concluded that the results of this study will serve as an up-to-date resource for coaches and swimmers working with elite breaststroke swimmers and as a useful insight to current underwater pullout trends.

## Introduction

In competitive swimming, rules and regulations associated with performing starts and turns have evolved over the decades and are governed by Fédération Internationale de Natation (FINA). A significant amendment in 2005 to the breaststroke event, permitted the inclusion of one butterfly kick during the underwater breaststroke pullout following a start or turn. A pullout is defined as the period from toe immersion following the start, or toe-off at the turn wall, until the swimmer breaks the water surface to commence free breaststroke swimming. After subsequent modifications, the current FINA (2017) SW 7.1. ruling states that: “After the start and after each turn, the swimmer may take one arm stroke completely back to the legs during which the swimmer may be submerged. At any time prior to the first breaststroke kick after the start and after each turn a single butterfly kick is permitted.” This latest iteration has resulted in various emerging techniques or movement pattern sequencing of the underwater breaststroke pullout phase, as swimmers aim to determine how best to utilize the butterfly kick (if at all). Considering the rules as prescribed by FINA, swimmers typically execute the underwater breaststroke pullout in the following manner: (1) passive glide with arms outstretched in a streamlined position overhead, (2) perform a pullout action of the arms so that they are extended at the sides of the trunk, (3) recovery of the arms and breaststroke kick toward breaking the surface, (4) that one butterfly kick takes place sometime before the breaststroke kick. It has been observed anecdotally that the placement of the butterfly kick relative to the pullout arm action varies across swimmers. It has been suggested that altering the placement of the kick may have consequences on the physiological demands of the underwater phase, the swimmers body alignment and consequently, resistive drag (McCabe et al., 2012). However, to date, no study within a competition setting has examined the technique trends displayed by elite swimmers or sought to assess the effectiveness of each technique throughout the underwater pullout following a breaststroke start or turn.

The underwater phase has been identified as the most important determinant of start and turn performance, as this is when the swimmer is traveling fastest through the water (Guimaraes and Hay, 1985; Seifert et al., 2007a; Connaboy et al., 2010; Tor et al., 2014a,b,c, 2015). Marinho et al. (2020) examined the underwater characteristics of the breaststroke pullout and reported that elite swimmers tend to spend longer (males 18.24%, females 16.85%), travel further (males 13.10%, females 11.94%), but are slower (males 4.43%, females, 4.03%) in the 200 m breaststroke underwater phases compared to the 100 m event. At the 2013 World Long Course Championships, Veiga and Roig (2016) reported that faster swimmers competing in the 100 m breaststroke, traveled with a faster underwater velocity (not further) compared to slower swimmers. More recently, Gonjo and Olstad (2021) reported that male elite swimmers displayed a faster mean glide velocity after both breaststroke starts and turns compared to sub-elite swimmers during a 100 m short-course time trial performance. On the basis that a faster underwater velocity is important in start and turn performance, researchers have recommended that coaches and swimmers should aim to optimize the underwater phase by executing a “good kinematical organization” and sequencing of the underwater breaststroke pullout movements in the most efficient way possible, i.e. by maximizing propulsion and minimizing resistive drag (Seifert et al., 2007b; Olstad et al., 2020; Sánchez et al., 2021).

The purpose of this study was to (1) ascertain the breaststroke pullout technique trends, as determined by the location of the fly-kick placement, across a range of international competitions, and (2) to assess the effectiveness of these pullout techniques as utilized by elite male and female swimmers across all competitive breaststroke events. It is hypothesized that a range of pullout techniques will be observed across swimmers and based on the findings of previous experimental studies (McCabe et al., 2012; Olstad et al., 2021; Seifert et al., 2021), there will be no significant difference in terms of performance across all breaststroke pullout techniques.

## Materials and methods

### Participants

Athletes competing in long-course breaststroke final races from World Championships 2015, 2017, 2019 and Olympics 2016 were included in the dataset. This resulted in a sample of 60 swimmers (*n* = 26 male, *n* = 34 female) across the 50 m (male = 26.79 ± 0.34 s, female = 30.25 ± 0.48 s), 100 m (male = 58.87 ± 0.69 s, female = 66.23 ± 0.90 s) and 200 m (male = 128.07 ± 1.00 s, female = 142.71 ± 1.43 s) breaststroke events. This totalled 150 race entries across the 50 m (*n* = 22 male, *n* = 18 female), 100 m (*n* = 26 male, *n* = 28 female) and 200 m (*n* = 26 male, *n* = 30 female) that were analyzed for this study. All the swimmers specialized in breaststroke, and were classified as elite based on the FINA points ‘Level 1' qualifying standards (≥875 pts) set by Ruiz-Navarro et al. (2022).

### Race analysis

**Figure 1:**
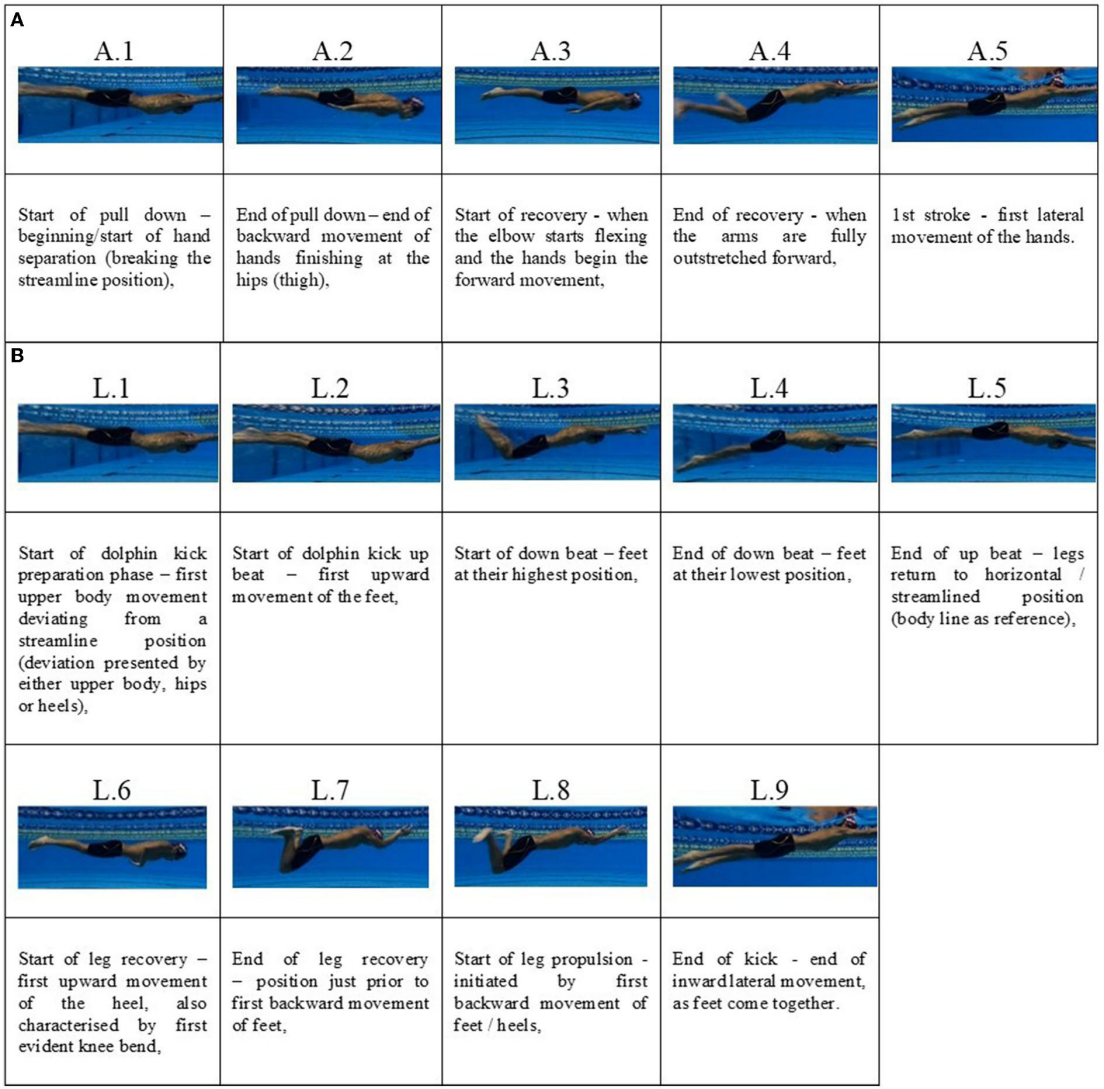
**(A)** Identification of key movement positions during the breaststroke underwater pullout phase with respect to the arm actions (A.1 to A.5) [A = arm]. **(B)** Identification of key movement positions during the breaststroke underwater pullout phase with respect to the leg actions (L.1 to L.9) [L = leg].

Following ethical approval from Manchester Metropolitan University, British Swimming's bespoke race analysis system, “Nemo” (Sheffield Hallam University), was used for all competition analysis. The system comprised of a single side-on panning Panasonic HC 1500 camera (resolution: 1920x1080, sampling frequency: 50Hz, shutter speed: 1/125-1/180 s), mounted at the highest point possible within the respective venues, usually around the 25 m mark of a 50 m pool to record all swim races. This experimental set-up is typical within a high-performance race analysis competition setting, demonstrating the study's ecologically valid approach (Nicol et al., 2021). To assess the validity of technique identification using a single above-water camera at 25 m, one analyst performed a pilot study analyzing 45 breaststroke starts (independent trials with respect to the current study) comparing technique identification between underwater footage and the single above-water camera approach used in this study. Temporal data corresponding to the key movement positions were identified and recorded (Figure 1). These positions are based on the actions performed by the arms and legs independently during the underwater pullout phase whilst adhering to FINA's regulations. A high intraclass correlation (ICC) was reported (ICC = 0.97; p <0.05), evidencing that this method has excellent agreement with respect to an underwater camera approach.

Similar to previous studies, start time was defined as the duration between the start signal to when the middle of the swimmer's head (goggle line) reached 15 m (Thompson et al., 2000; Cossor and Mason, 2001; Veiga et al., 2013; Tor et al., 2014a,b; Marinho et al., 2020). The start signal was identified as the first frame where the strobe light was visible and is the point at which the video is synchronized. Turn time was defined as the time from when the swimmer's hands touch the wall to the head reaching 15 m out from the wall. The rationale for this definition was to isolate the turn/underwater pullout performance and eliminate any possible influence of the swimmer's approach to the wall.

### Determination of underwater breaststroke pullout techniques

To determine the temporal sequencing and the techniques associated with the breaststroke underwater pullout phase, each of the races were firstly visually inspected to identify key movement positions (Figure 1).

The identification of these key movement positions facilitated the classification of the following eight phases throughout the underwater pullout:

1) 1st glide (A.1)—from toe immersion (dive start) / toes having left the wall (turn exit) to first movement deviating from a streamline position (common instances of deviation: hand separation/start of dolphin kick preparation phase/first upper body movement).
2) Dolphin kick (L.1 to L.5).
3) Pull down (A.1 to A.3)—time between beginning of hand separation (breaking the streamline position) to the end of pull down—end of backward movement of hands finishing at the hips (or thighs).
4) 2nd glide (A.3)—time from end of pull down to the start of arm recovery—as the elbow starts flexing and the hands start forward movement.
5) Arm recovery (A.3 to A.4)—time between start of arm recovery to the instance when the arms are fully extended.
6) Leg recovery (L.6 to L.7)—time between first initiation of knee flexion, to position just prior to first backward movement of feet.
7) Propulsive kick (L.8 to L.9)—time between first backward movement of feet, to end of kick—as the feet come together ending inward lateral movement.
8) 3rd glide (A.5 and L.9)—time between feet coming together and first lateral movement of hands or as the head breaks water surface.

**Figure 2:**
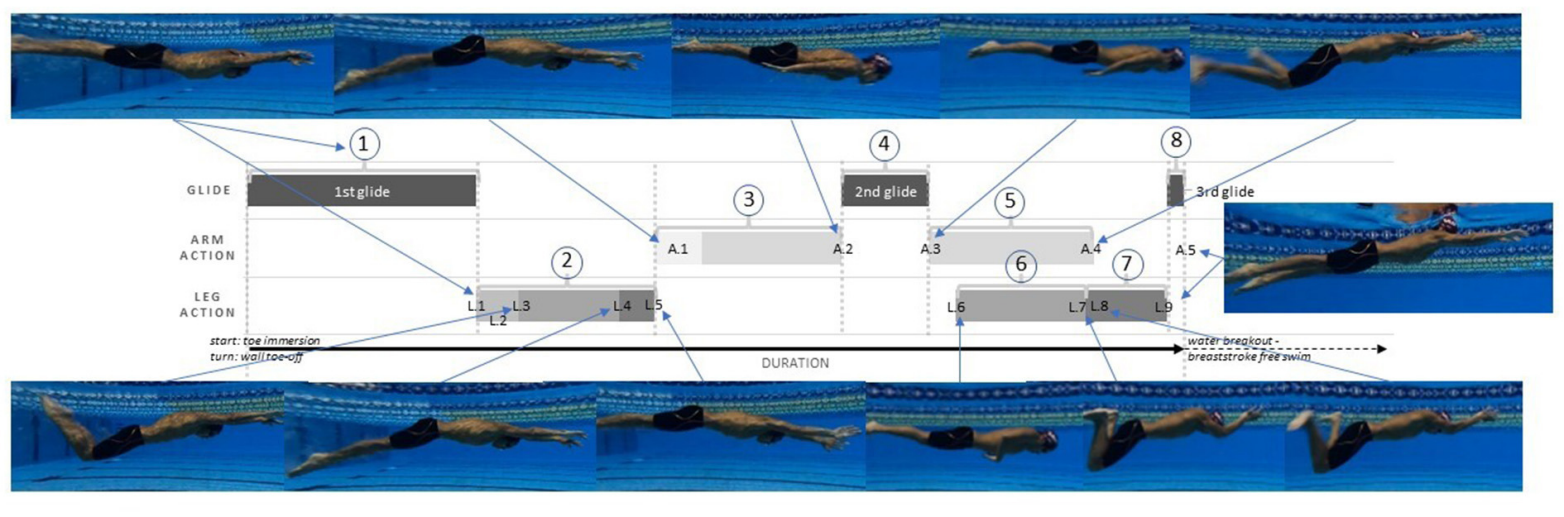
A phase duration sequence of the Fly-Kick First Technique. Refer to Figures 1A,B for number and letter annotation information.

Once the phases were defined, the underwater pullout technique could be determined based on the temporal order in which the phases were executed. For example, Figure 2 presents a graphical representation of the interplay between the arm (A.1–A.5) and leg actions (L.1–L.9), connected by three defined glide phases through the duration of the pullout in breaststroke swimming. The sequenced movement pattern observed in Figure 2, will be from hereon referred to as the “Fly-Kick First” technique whereby the fly-kick is initiated and completed prior to the arm pull-down. A key feature of the Fly-Kick First technique is that there is a clear separation/takeover from one action to the next (arms and legs) throughout the underwater pullout.

**Figure 3:**
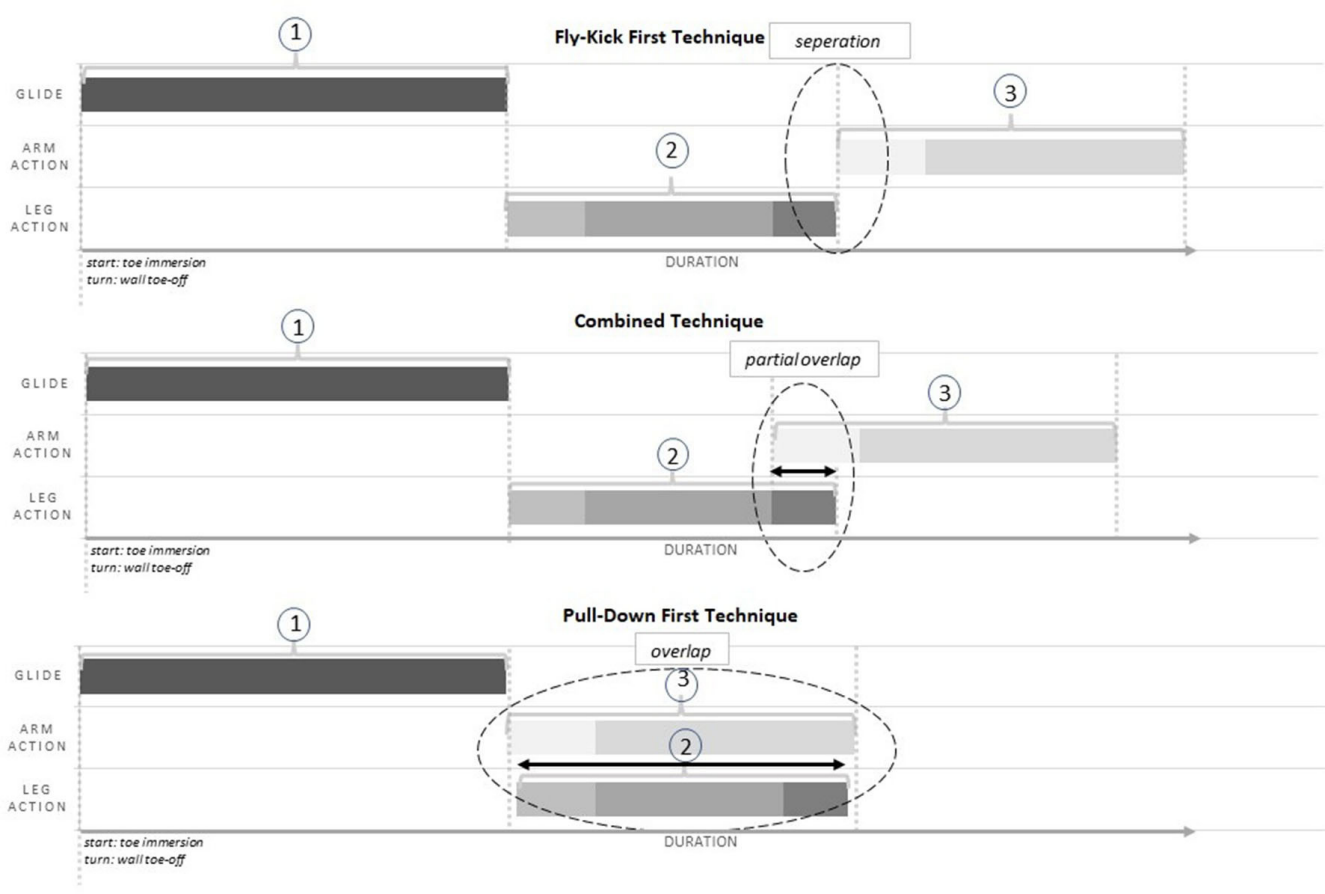
Breaststroke pullout phase profile variations; indicating the primary difference in arm and leg action sequence across the Fly-Kick First, the Combined and the Pull-Down First techniques. The numbers 1, 2, and 3 represent the first three phases of the pullout [1st Glide, Dolphin Kick, and Pull Down phase].

Through observation of all 150 trials captured across the four competitions within this study, two further technical trends were identified and categorized, relative to the Fly-Kick First technique (Figure 3). The “Combined” technique is characterized by the initiation of the pullout prior to the completion of the fly-kick; consequently a degree of partial overlap between the arm and leg actions is observed. Finally, the “Pull-Down First” is distinguishable due to the arms fully pulling down to the sides of the trunk prior to the completion of the fly-kick. The Pull-Down First technique is unique in that it is the only movement pattern whereby the arms are initiated prior to any leg action.

In defining the three techniques across the participants in this study; a visual representation provided below highlights the primary temporal variation in arm and leg action sequencing during the pullout progression across all techniques. The below representations exclude all actions occurring after the arm action “A.2” (i.e. prior to the 2nd glide), in order to place a greater emphasis on the underwater temporal phase variation between the three techniques.

### Statistical analysis

Following key underwater position identification (Figure 1), each swimmer was categorized into a ‘technique' sub-group. Descriptive statistics for each technique, such as time to complete the start and turn per racing event were reported across the combined sample and also separated with respect to gender. Homogeneity of variance was assessed using the Levene's test, before a one-way ANOVA was performed to determine any significant differences between pullout techniques. A Tukey *post-hoc* correction was used to assess the differences between underwater pullout techniques. The significance level across all statistical tests was set at p < 0.05. The eta square (η^2^) was used to assess the magnitude of the effect size, with: (i) without effect if 0 < η^2^ ≤ 0.04; (ii) minimum if 0.04 < η^2^ ≤ 0.25; (iii) moderate if 0.25 < η^2^ ≤ 0.64; and (iv) strong if η^2^ > 0.64 (Ferguson, 2009). All statistical analysis was conducted using SPSS version 27.0 (Statistical Package for Social Sciences, IBM Corp. Armonk, NY, USA).

## Results

A summary of the descriptive statistics calculated for each technique across all race distances is displayed within Table 1. It was found that the most common underwater pullout technique utilized by elite competitive breaststroke swimmers following a start and turn was the Combined technique (total observations = 71), followed by the Fly-Kick First technique (total observations = 65) and the Pull-Down First technique (total observations = 14). There was no statistical difference found between techniques across the 50m (start: p = 0.41, η^2^ = 0.05), 100m (start: p = 0.06, η^2^ = 0.11; turn: p = 0.43, η^2^ = 0.30) or 200m (start: p = 0.62, η^2^ = 0.02; turn: p = 0.74, η^2^ = 0.01) breaststroke race events.

**Table 1:** Descriptive statistics for start and turn performances for each of the underwater pullout techniques across the 50, 100, and 200 m breaststroke race events.

**Technique**	**Start 50 m (s)**	**Count**	**Start 100 m (s)**	**Turn 100 m (s)**	**Count**	**Start 200 m (s)**	**Turn 200 m (s)**	**Count**
Fly-Kick First	6.80 ± 0.57	16	7.08 ± 0.68	9.45 ± 0.58	26	7.35 ± 0.70	29.24 ± 21.40	23
Combined	6.67 ± 0.59	20	6.84 ± 0.61	9.34 ± 0.62	22	7.19 ± 0.74	29.36 ± 2.08	29
Pull-Down First	7.11 ± 0.75	4	7.56 ± 0.57	9.68 ± 0.41	6	7.47 ± 0.76	30.18 ± 2.17	4
Avg.	6.77 ± 0.60		7.03 ± 0.67	9.43 ± 0.58		7.28 ± 0.71	29.37 ± 2.19	

This dataset includes all male and female performances combined.

**Figure 4:**
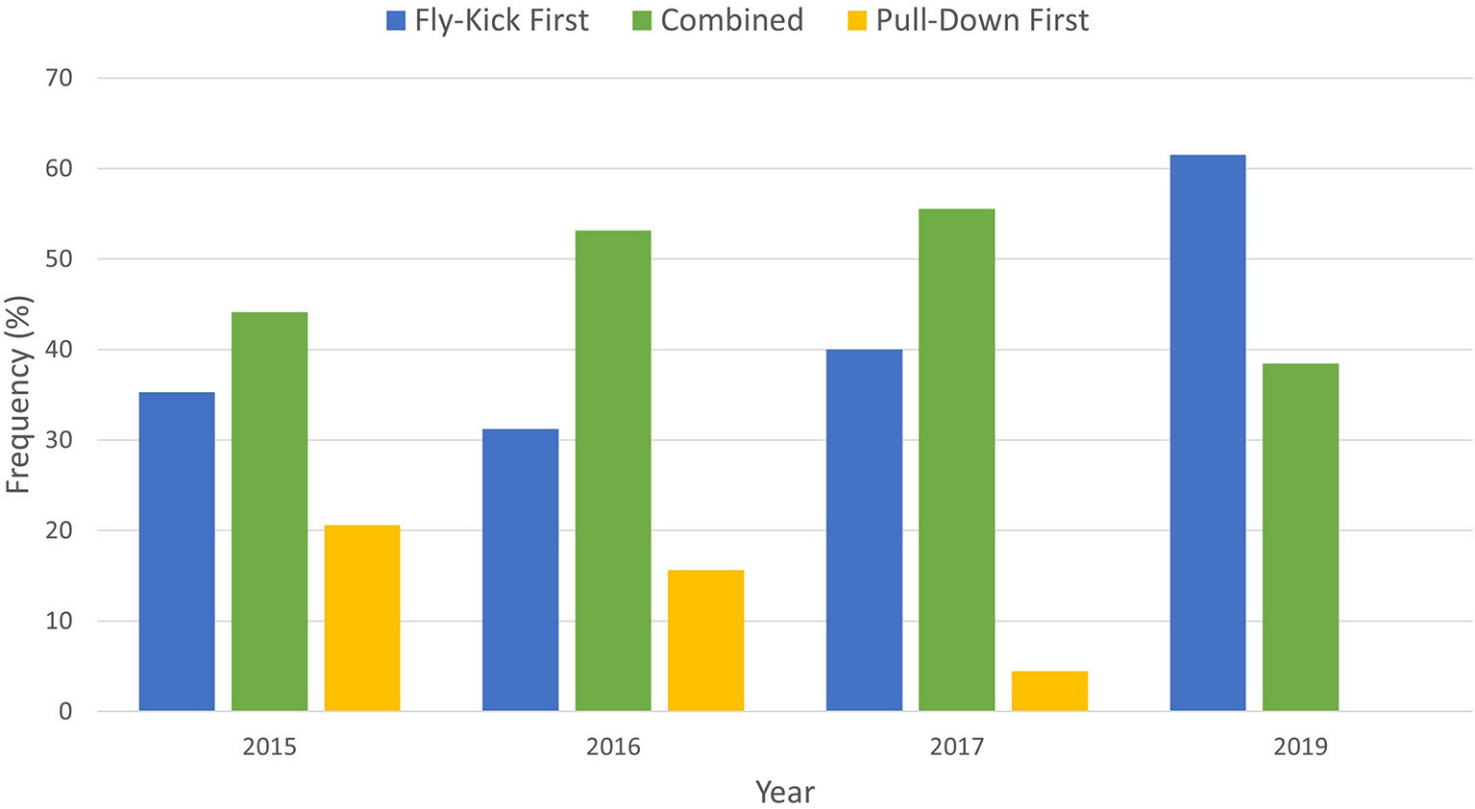
Underwater breaststroke pullout technique trends for elite swimmers during the period 2015–2019.

Figure 4 illustrates the underwater breaststroke pullout technical trends by elite swimmers competing across major competitions 2015–2019. It is observed that the Fly-Kick First technique has increased in popularity over the years, whilst the Pull-Down First technique has progressively decreased in popularity. Throughout the data collection period, the Combined technique appeared to be the most favored underwater pullout technique until 2019, when the Fly-Kick First was observed to be executed most often during the World Championships.

**Figure 5:**
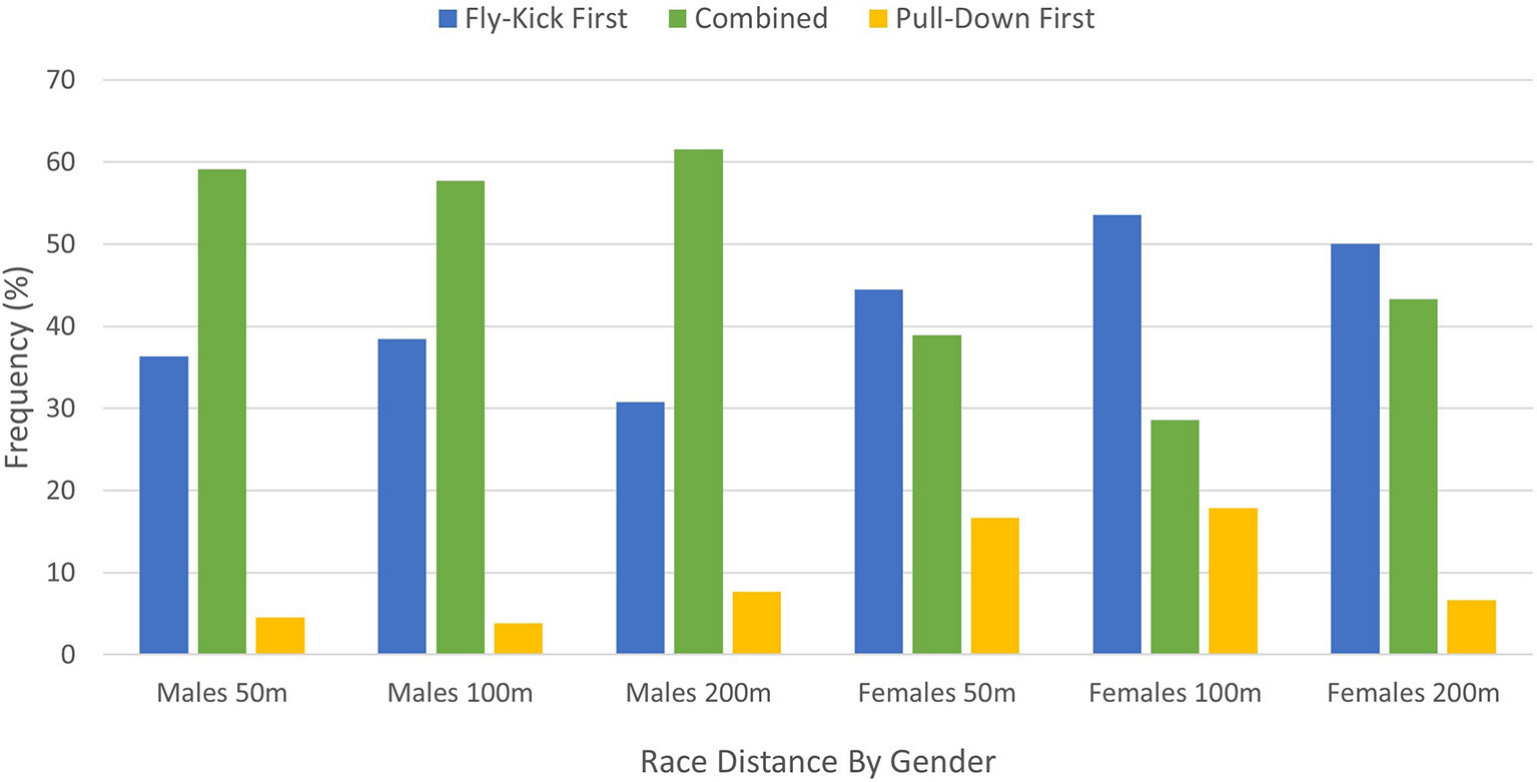
Underwater breaststroke pullout trends across all race distances for male and female elite swimmers.

Tables 2, 3 provide an overview of the start and turn performances for each of the underwater pullout techniques across the 50 m, 100 m, and 200 m breaststroke races events for male and female swimmers respectively. Figure 5 illustrates that male elite breaststroke swimmers tend to favor the Combined technique, followed by the Fly-Kick First technique across all race events. Alternatively, it was observed that elite female breaststroke swimmers tend to favor the Fly-Kick First closely followed by the Combined technique. Statistical analysis revealed there were no significant differences between techniques across the race distances for either male (Table 2) or female (Table 3) swimmers, with one exception. Within the 100 m female event, a significant difference (p=0.05) was found between techniques when turning. *Post hoc* results indicated a difference between the Combined technique and Pull-Down First technique (*p* = 0.05).

**Table 2:** Male start and turn performances for each of the underwater pullout techniques across the 50, 100, and 200 m breaststroke race events.

**Technique**	**Male start 50 m (s)**	**Male start 100 m (s)**	**Male turn 100 m (s)**	**Male start 200 m (s)**	**Male turn 200 m (s)**
Fly-Kick First	6.27 ± 0.16	6.36 ± 0.28	8.83 ± 0.13	6.48 ± 0.20	26.63 ± 10.61
Combined	6.26 ± 0.15	6.41 ± 0.19	8.92 ± 0.30	6.56 ± 0.20	27.67 ± 1.02
Pull-Down First	6.04	6.52	8.90	6.82 ± 0.14	28.36 ± 0.37
Avg.	6.19 ± 0.15	6.39 ± 0.23	8.88 ± 0.23	6.55 ± 0.21	27.40 ± 1.29
Significance (p)	0.38	0.75	0.72	0.11	0.09
Effect size (η^2^)	0.10	0.03	0.03	0.18	0.19

**Table 3:** Female start and turn performances for each of the underwater pullout techniques across the 50, 100, and 200 m breaststroke race events.

**Technique**	**Female start 50 m (s)**	**Female start 100 m (s)**	**Female turn 100 m (s)**	**Female start 200 m (s)**	**Female turn 200 m (s)**
Fly-Kick First	7.33 ± 0.14	7.61 ± 0.27	9.94 ± 0.18	7.82 ± 0.24	30.63 ± 1.33
Combined	7.43 ± 0.15	7.60 ± 0.18	10.08 ± 0.14	7.97 ± 0.16	31.44 ± 0.53
Pull-Down First	7.46 ± 0.30	7.77 ± 0.28	9.84 ± 0.17	8.12 ± 0.11	32.01 ± 0.72
Avg.	7.41 ± 0.20	7.63 ± 0.25	9.96 ± 0.18	7.90 ± 0.22	31.08 ± 10.10
Significance (p)	0.42	0.41	0.05^*^	0.06	0.07
Effect Size (η^2^)	0.11	0.07	0.22	0.19	0.18

* Significant difference p < 0.05.

Observationally, it was noted that nine swimmers changed their underwater pullout technique during the 200 m race event, and three swimmers modified their pullout technique in the 100 m for the start vs. turn. The implications of this observation will be further explored within the discussion.

## Discussion

The purpose of this study was to characterize the breaststroke underwater pullout technique trends utilized by elite swimmers within a competition setting across all race events and to assess the effectiveness of each. The context underpinning this study was to consider the inclusion of the fly-kick during the underwater pullout phase as a consequence of (FINA, 2017) SW 7.1 regulatory modification. A unique aspect of this current study was access to a large dataset of elite swimmers which was captured and analyzed using the same methodological approach across four international competitions, including World Championships and Olympic Games, ensuring the capability to provide a rigorous and broad characterization of the underwater breaststroke pullout techniques by elite male and female swimmers.

Using footage from key international competitions, three different pullout techniques were identified in this study: the Fly-Kick First technique (fly-kick is initiated and completed prior to pull-down), the Combined technique (pull-down is initiated before the fly-kick is complete, consequently an overlap of phases is observed) and the Pull-Down First technique (pull-down is completed prior to fly-kick). Therefore, our first hypothesis that a range of pullout techniques will be observed across swimmers was accepted. These three techniques differ slightly with respect to Seifert et al. (2021) who identified three coordination profiles, namely: “Continuity”, “Glide” and “Superposition”. They defined the Continuity profile as the synchronization of the arm pull-down beginning as the fly-kick ends, which is similar to the Fly-Kick First technique as described in the current study. The Glide profile was defined as the initiation of the arm pull-down following a glide phase post completion of the fly-kick. This coordination profile was incorporated within the Fly-Kick First technique in this study, perhaps as a result of no supporting underwater video footage and thus inability to identify a distinct glide portion following the fly-kick completion. The Combined technique and Seifert et al. (2021) Superposition profile are similar in that both identify an overlap of the arm pull-down and completion of the fly-kick. In addition, this study uniquely observed and identified the Pull-Down First technique which was not evident within the Seifert et al. (2021) study. The variation in underwater pullout technique identification between studies may be due to the data being captured in two different environments (research vs. competition setting). The research-based technique identification by Seifert et al. (2021) was conducted using underwater cameras which would have increased the visibility of key points compared to using an above-water camera in the current study. Undoubtedly, underwater footage is beneficial to accurately track key positions associated with the underwater breaststroke pullout. However, this study did report high validity (ICC = 0.97) in terms of the current methodological approach, thus providing confidence with respect to the dataset obtained. Moreover, it should be highlighted that international competitions restrict the placement of underwater cameras, meaning that above-water camera systems are typically utilized to perform competition race analysis. Therefore it is acknowledged that the method used to obtain data in this study allows direct comparison with the existing literature, whilst also providing an ecologically valid approach that is meaningful to swimmers and coaches in the context of trends and techniques used within a competition environment. Other considerations to explain the differences in technique identification between Seifert et al. (2021) and the current study may be due to the variation of swimmers sampled in terms of magnitude (n = 14 vs. n = 60) gender (n = 14 males vs. n = 26 males & n = 34 females) and performance level (64.42 ± 3.11 s for 100 m short course vs. males = 58.87 ± 0.69 s, females = 66.23 ± 0.90 s for 100 m long course). Indeed, Seifert et al. (2007b) and Veiga et al. (2014) both reported that the underwater swimming phases differed significantly with respect to expertise, with competitors tending to organize the underwater portion of the race according to the swimmer's skill level which may account for the differences between previous studies and the current one.

The results of this study show that across all race distances, the most common underwater pullout technique utilized by elite competitive breaststroke swimmers (male and female) following a start and turn was the Combined technique, followed by the Fly-Kick First technique and the Pull-Down First technique respectively. This differs from Seifert et al. (2021) who found that based on their population, the Continuity profile (the Fly-Kick First Technique) was more popular followed by the Superposition profile (the Combined Technique). As discussed previously, it is possible that skill level, gender and the length of the pool (short vs. long course), may all be contributing factors that influence the style of underwater technique utilized which requires further investigation. Another consideration is based on the observations in this study that the underwater pullout temporal movement sequences have evolved over the years (Figure 4). Although the Combined technique tended to be favored by swimmers across the period 2015–2017, this was superseded by the Fly-Kick First technique in 2019. Therefore, in agreement with Seifert et al. (2021), there are qualitative indications that the Fly-Kick First technique has become most popular in recent years. Continuous competition monitoring is required to confirm this observation; however, it is also possible that elite swimmers are still experimenting with the fly-kick placement to optimize their individual underwater performance.

Interestingly, when the dataset was filtered by gender, it was observed that male and female elite breaststroke swimmers tended to favor different techniques during the underwater pullout phase. Male swimmers were observed to utilize the Combined technique most often followed by the Fly-Kick First technique across all race distances, whereas female swimmers favored the Fly-Kick First technique followed by the Combined technique. It has previously been established that body morphology directly affects a swimmer's hydrodynamic resistance, with the majority of literature suggesting that males experience increased drag compared to females due to differences in body shape (Toussaint et al., 1988). Vilas-Boas et al. (2010) reported that during an underwater breaststroke sequence, females tended to experience lower drag values during the first gliding position (arms overhead in a streamlined position) compared to their male counterparts. In the second gliding position (arms extended by the swimmer's sides) males experienced lower drag values compared to females. The authors suggested that these differences in drag values were linked to differences in cross sectional area, body length and slenderness between the genders. It is therefore possible to extrapolate that the observed differences between genders in terms of favored underwater breaststroke pullout techniques in this study may be due to differences in anthropometry and morphology, thus the hydrodynamic resistance experienced. Although additional investigation is warranted to confirm such associations, a strong “take-home message” from this study is that coaches should not prescribe the same technique across genders.

Our second hypothesis is accepted as when swimmers were combined, this study did not find any significant difference in start or turn performance in relation to the technique used and effect sizes were reported as moderate-small. This is in agreement with McCabe et al. (2012), Seifert et al. (2021), and Olstad et al. (2021) and who all reported similar underwater performance outcomes could be achieved irrespective of the technique used and that the selected technique may be due to individual preference. When examining genders independently, this study found that female swimmers competing in the 100 m event, were 0.24 s or 2% faster using the Pull-Down First compared to the Combined technique. This is interesting in the context of the Pull-Down First technique popularity progressively declining over the years, suggesting that trends or techniques favored by elite swimmers may not always be the most effective techniques to adopt. It is concluded that based on the overall results of this research, no technique appears to be more effective than the other. Rather, the technical choice appears to be driven by individual preference, which may be influenced by anthropometric or morphology factors. If the swimmer can execute the chosen technique effectively, they should be competitive amongst their peers, but this may require experimentation within a training environment.

Another observation found from the qualitative pullout analysis showed that some elite swimmers altered their pullout technique between the start and the turn. There were nine swims in the 200 m races and three swims in the 100 m race where athletes changed their technique. Interestingly, Seifert et al. (2021) also observed that two swimmers changed the way they synchronized their fly-kick and arm pullout between the start and three turns. The statistical analysis conducted in this study was completed based on the technique that the swimmers utilized in the start. Hence, this was a limitation of the study and future studies of this nature should account for this change to reveal further trends amongst elite breaststrokers and perhaps explore why this may occur.

Considering the methodological limitations of this study, the results should be interpreted cautiously until future investigations confirm our technique observations *via* capturing underwater footage (either in a competition or experimental setting). Regardless, this is the first paper to qualitatively report the underwater breaststroke pullout technical trends across multiple competitions and thus provides a novel contribution to the research area and swimming community. Future studies should explore the underwater pullout trends further in terms of spatiotemporal characteristics and examine the potential discriminant factors associated with performing these different techniques. For example, it would be useful to quantify the break-out distances associated with each technique to assess if the time to 15 m was influenced by surface swimming, rather than the differing pull-out techniques. It is also recommended that the velocity profiles of each pullout technique be investigated. As the breaststroke pullout follows the dive and wall push-off phases within a race lap; the primary aim of the pullout should be to maintain the speed generated following these phases. This is achieved by reducing drag in optimizing body form and minimizing velocity degradation through beneficial use of leg and arm actions prior to the free-swimming phase. Velocity profiles could allow for a greater understanding of each of the techniques in relation to these instances and facilitate coaches to make more informed decisions on technique selection for optimizing start and turn performance.

### Practical applications

The findings from this study will yield multiple practical implications for coaches and sport scientists. Given the significant contribution of the start and turn in breaststroke performance at the elite level, large importance should be placed on the pullout phase during training practices. This study demonstrates that technique selection is largely individual, but coaches should conduct appropriate biomechanical testing to ensure that the fastest technique is being used for each individual's start and turn performance. The technique used should also consider the physiological cost, as the selection of the Combined technique and Pull-Down First technique may influence the breakout distance (as reported by McCabe et al., 2012), which would have implications on the number of strokes to be taken during the free-swimming phase. Further research surrounding the breakout distances of each pullout technique will enhance the understanding of the underwater phase in breaststroke specific events.

## Conclusion

This was the first study of its kind to provide an in-depth analysis focusing solely on the breaststroke pullout using a large cohort of elite swimmers during competition. This study identified three common breaststroke pullout techniques used by elite swimmers during multiple key international competitions. Based on qualitative observations, the most frequent pullout technique was the Combined technique, with indications this was changing toward a Fly-Kick First technique preference in recent years. Male and female swimmers appear to utilize different underwater pullout techniques; therefore, it is recommended they should not be coached adhering to the same technical model. Although there was no difference in performance across techniques, it is important that swimmers are proficient in their chosen technique. The results from this study will serve as a resource for coaches and swimmers working with breaststroke swimmers competing at the highest international level.

## Data availability statement

The raw data supporting the conclusions of this article will be made available by the authors, without undue reservation.

## Ethics statement

The studies involving human participants were reviewed and approved by Manchester Metropolitan University Ethics Committee. Written informed consent for participation was not required for this study in accordance with the national legislation and the institutional requirements.

## Author contributions

CM and ET conceived and designed the study. EM performed all data analysis. RH produced the Figures. CM, ET, and EM carried out the drafting of the manuscript. All authors reviewed the manuscript and approved the submitted version.

## Conflict of interest

Author ET was employed by Forethought Pty Ltd. The remaining authors declare that the research was conducted in the absence of any commercial or financial relationships that could be construed as a potential conflict of interest.

## Publisher's note

All claims expressed in this article are solely those of the authors and do not necessarily represent those of their affiliated organizations, or those of the publisher, the editors and the reviewers. Any product that may be evaluated in this article, or claim that may be made by its manufacturer, is not guaranteed or endorsed by the publisher.

## References

[B1] Connaboy C. Coleman S. Moir G. Sanders R. (2010). Measures of reliability in the kinematics of maximal undulatory underwater swimming. Med. Sci. Sports Exerc. 24, 762–770. 10.1249/MSS.0b013e3181badc68 19952849

[B2] Cossor J. M. Mason B. R. (2001). Swim start performances at the Sydney 2000 Olympic Games. San Francisco, CA: Paper presented at the International Synopsium of Biomechanics in Sports.

[B3] Ferguson C. J. (2009). An effect size primer: a guide for clinicians and researchers. Prof. Psychol. Res. Pr. 40, 532–538. 10.1037/a0015808

[B4] FINA. (2017). FINA Swimming Rules 2017 - 2021. Available online at: http://www.fina.org/sites/default/files/2017_2021_swimming_16032018.pdf (accessed May 09, 2022).

[B5] Gonjo T. Olstad B. H. (2021). Race analysis in competitive swimming: a narrative review. Int. J. Environ. Res. Public Health 18, 69. 10.3390/ijerph18010069 33374118 PMC7795652

[B6] Guimaraes A. C. S. Hay J. G. (1985). A mechanical analysis of the grab starting technique in swimming. J. Appl. Biomech. 1, 25–35. 10.1123/ijsb.1.1.2525978370

[B7] Marinho D. A. Barbosa T. M. Neiva H. P. Silva A. J. Morais J. E. (2020). Comparison of the start, turn and finish performance of elite swimmers in 100 m and 200 m races. J. Sports Sci. Med. 19, 397. 32390734 PMC7196746

[B8] McCabe C. Mason B. Fowlie J. (2012). A temporal investigation into the butterfly kick placement following a breaststroke start and turn. Melbourne: Paper presented at the 30th Conference of the International Society of Biomechanics in Sport.

[B9] Nicol E. Adani N. Lin B. Tor E. (2021). The temporal analysis of elite breaststroke swimming during competition. Sports Biomechanics. 1–13. 10.1080/14763141.2021.1975810 34547991

[B10] Olstad B. H. Gonjo T. Conceição A. Štastn,ý J. Seifert L. (2021). Arm–leg coordination during the underwater pull-out sequence in the 50, 100 and 200 m breaststroke start. J. Sci. Med. Sport. 12 10.1016/j.jsams.2021.08.006 34462220

[B11] Olstad B. H. Wathne H. Gonjo T. (2020). Key factors related to short course 100 m breaststroke performance. Int. J. Environ. Res. Public Health. 17, 6257. 10.3390/ijerph17176257 32867383 PMC7503867

[B12] Ruiz-Navarro J. J. Lopez-Belmonte O. Gay A. Cuenca-Fernandez F. Arellano R. (2022). A new model of performance classification to standardise the research results in swimming. Eur. J. Sport Sci. 20, 1–11. 10.1080/17461391.2022.2046174 35193458

[B13] Sánchez L. Arellano R. Cuenca-Fernández F. (2021). Analysis and influence of the underwater phase of breaststroke on short-course 50 and 100m performance. Int. J. Perform. Anal. 1–17. 10.1080/24748668.2021.1885838

[B14] Seifert L. Chollet D. Chatard J. C. (2007a). Kinematic changes during a 100-m front crawl: effects of performance level and gender. Med. Sci. Sports Exerc. 39, 1784–1793. 10.1249/mss.0b013e3180f62f38 17909406

[B15] Seifert L. Conceição A. Gonjo T. Štastn,ý J. Olstad B. H. (2021). Arm – Leg coordination profiling during the dolphin kick and the arm pull-out in elite breaststrokers. J. Sports Sci. 1–9. 10.1080/02640414.2021.1950446 34878366

[B16] Seifert L. Vantorre J. Chollet D. (2007b). Biomechanical analysis of the breaststroke start. Int. J. Sports Med. 28, 970–976. 10.1055/s-2007-965005 17497572

[B17] Thompson K. G. Haljand R. MacLaren D. P. (2000). An analysis of selected kinematic variables in national and elite male and female 100-m and 200-m breaststroke swimmers. J. Sports Sci. 18, 421–431. 10.1080/02640410050074359 10902677

[B18] Tor E. Pease D. Ball K. (2014a). Characteristics of an elite swimming start. Canberra: Paper presented at the Biomechanics and Medicine in Swimming Conference 2014.

[B19] Tor E. Pease D. Ball K. (2014b). Comparing three underwater trajectories of the swimming start. J. Sci. Med. Sport. 18, 725–729. 10.1016/j.jsams.2014.10.005 25455956

[B20] Tor E. Pease D. Ball K. (2015). Key parameters of the swimming start and their relationship to start performance. J. Sport Sci. 33, 1313. 10.1080/02640414.2014.990486 25555171

[B21] Tor E. Pease D. Ball K. Hopkins W. G. (2014c). Monitoring the effect of race-analysis parameters on performance in elite swimmers. Int. J. Sports Physiol. Perform. 9, 633–636. 10.1123/ijspp.2013-0205 24155134

[B22] Toussaint H. M. de Groot G. Savelberg H. H. C. M. Vervoorn K. Hollander A. P. van Ingen Schenau G. J. (1988). Active drag related to velocity in male and female swimmers. J. Biomech. 21, 435–438. 10.1016/0021-9290(88)90149-2 3417695

[B23] Veiga S. Cala A. Mallo J. Navarro E. (2013). A new procedure for race analysis in swimming based on individual distance measurements. J. Sports Sci. 31, 159–165. 10.1080/02640414.2012.723130 22989356

[B24] Veiga S. Mallo J. Navandar A. Navarro E. (2014). Effects of different swimming race constraints on turning movements. Hum. Mov. Sci. 36, 217–226. 10.1016/j.humov.2014.04.002 24875044

[B25] Veiga S. Roig A. (2016). Underwater and surface strategies of 200 m world level swimmers. J. Sports Sci. 34, 766–771. 10.1080/02640414.2015.1069382 26186108

[B26] Vilas-Boas J. P. Costa L. Fernandes R. J. Ribeiro J. Figueiredo P. Marinho D.. (2010). Determination of the drag coefficient during the first and second gliding positions of the breaststroke underwater stroke. J. Appl. Biomech. 26, 324–331 10.1123/jab.26.3.324 20841624

